# Polyethylene glycol‐based ultrasonic‐assisted enzymatic extraction, characterization, and antioxidant activity in vitro and in vivo of polysaccharides from *Lonicerae japonica* leaves

**DOI:** 10.1002/fsn3.1186

**Published:** 2019-09-13

**Authors:** Wei Wu, Tingrong Huang, Fu Xiang

**Affiliations:** ^1^ Hubei Key Laboratory of Economic Forest Germplasm Improvement and Resources Comprehensive Utilization Hubei Collaborative Innovation Center for the Characteristic Resources Exploitation of Dabie Mountains Huanggang Normal University Huangzhou China; ^2^ Edong Healthcare City Hospital of Traditional Chinese Medicine (Infectious Disease Hospital) Huangshi China

**Keywords:** antioxidant activity, *Lonicerae japonica* leaves, PEG‐based ultrasonic‐assisted enzymatic extraction, polysaccharides

## Abstract

In this paper, polyethylene glycol (PEG)‐based ultrasonic‐assisted enzymatic extraction (UAEE) was employed in polysaccharides extraction from *Lonicerae japonica* leaves (LJLP). The optimal extraction conditions (extraction time of 33 min, PEG concentration of 30%, and ultrasonic power of 191 W) were obtained by Box–Behnken design (BBD). Under this condition, the LJLP yield was 14.76%. Furthermore, LJLP was identified as a typical heteropolysaccharide considering the main constitutive monosaccharides include galactose (32.3%), glucose (20.9%), and ribose (15.2%). Moreover, LJLP exhibited high total reducing power and considerable scavenging activities on superoxide radicals, in a concentration‐dependent manner in vitro. In addition, antioxidant analysis in vivo revealed that for the LJLP‐treated mice, the superoxide dismutase (SOD), glutathione peroxidase (GSH‐Px), catalase (CAT) activities, and total antioxidant capacity (TAOC) were significantly increased, while the level of malondialdehyde (MDA) was decreased in both serum and liver.

## 1 INTRODUCTION

*Lonicera japonica* can be deciduous and evergreen shrubs, or climbers with twining stems. It is widely distributed in eastern Asia countries such as Japan, Korea, and China, which is also known as *Japanese honeysuckle* and *Jin Yin Hua* in these areas (Chen, Liou, Tzeng, Lee, & Liu, 2012). Previous studies revealed that *L. japonica *and its active principles not only have the functions of anti‐inflammatory, antibacterial, antiviral, and antiendotoxin but also help to reduce blood fat (Shang, Pan, Li, Miao, & Ding, 2011; Zhang, Yang, & Liu, 2008). Recently, many plant polysaccharides have been proved possessing varied biological activities, for example: improve the immune function of organism, antitumor, anti‐aging, anti‐inflammatory, antiradiation, hypoglycemic, and so on (Ferreira, Passos, Madureira, Vilanova, & Coimbra, 2015; Thambiraj, Phillips, Koyyalamudi, & Reddy, 2015; Wasser, 2002). Therefore, *L. japonica* polysaccharides have been attracted increasing attention. In China, *L. japonica* is planted extensively not only for its medicinal value but also for its function of ornamental plants. Most utilization of the major parts of this plant is mainly focused on flower buds, and the application of leaves is overlooked, causing colossal waste of the natural resources. Nowadays, the market prices of flower buds of *L. japonica* are on rising, and leaves of *L. japonica* will be a good alternative for the raw material of *L. japonica* leaves polysaccharides (LJLP), which have a lower price and richer sources. So it is requisite to study the exploitation and utilization of LJLP from leaves of *L. japonica*.

It is reported that the solvents for extraction of polysaccharides mainly include water, ethanol, alkaline solutions, and so on (Georgiadis et al., 2012; Liu, Liu, Zhang, & Zhang, 2012; Rout & Banerjee, 2007). Recently, it was first reported that polyethylene glycol (PEG) was applied to extract flavone and coumarin compounds from medicinal plants (Zhou, Xiao, Li, & Cai, 2011). As a new green solvent, PEG has a number of unique advantages, such as low flammability, biodegradability, and nonvolatility. The most important is that PEG has a good safety considering it has been classified as GRAS (Generally Recognized as Safe) for internal consumption by the US FDA (Zhou et al., 2011). In this study, PEG was chosen as a green solvent for extraction of LJLP from leaves of *L. japonica*.

In this study, extracting conditions, preliminary characterization, and antioxidant ability in vitro of LJLP were investigated. Firstly, Box–Behnken design (BBD) was applied to optimize the extraction process of LJLP from leaves of *L. japonica*. As an effective extractive method, ultrasound‐assisted extraction (UAE) has been investigated in the extraction of LJLP, and enzymes have been introduced to disrupt the plant cell walls, which help to enhance the extraction yields. After preparation through the extracting conditions, the crude LJLP was purified by ethanol precipitation. Then, the preliminary characterization of these polysaccharides was studied by high‐performance liquid chromatography (HPLC) and Fourier transform infrared spectroscopy (FT‐IR). Moreover, antioxidant activities of LJLP were also evaluated in vitro and in vivo.

## 2 MATERIALS AND METHODS

### 2.1 Materials and chemicals

*Lonicera Japonica* leaves were provided by Hubei Chutianshu Pharmaceutical Co., Ltd. The dried leaves were filtered by a 40 mesh screen after ground and set aside. The power was subjected to degrease by petroleum ether (60–90°C), 80% ethanol help to remove some potential impurities such as flavonoids, lipids, pigments, monosaccharides, oligosaccharides, and so on. The chemical reagents used in experiments were all analytically pure and purchased from Sinopharm Chemical Reagent Co., Ltd. Distilled water was used throughout the study.

### 2.2 Extraction of polysaccharides

In this study, ultrasound‐assisted extraction was performed in an ultrasonic‐microwave combined reaction system (Nanjing Atpio Instruments Manufacturer).

5.0 g prepared samples were immersed in 100 ml PEG aqueous solution, and enzyme solution (cellulose:pectase:trypsin was 2:2:1) was added to improve the efficiency of wall‐breaking. Through ultrasonic extraction, centrifugation (7104  *g*, 10 min), the supernatant was sufficiently mixed with 4 times volumes of anhydrous ethanol and stored at 4°C overnight. The precipitate was harvested by centrifugation and dried to gain the raw LJLP. The content of LJLP was determined by the phenol–sulfuric acid method with D‐glucose as the standard substance (Dubois, Gilles, Hamilton, Rebers, & Smith, 1956). The formula of extraction yield (%) of LJLP was as follows: LJLP yield (%) = LJLP weight (g)/sample weight (g).

### 2.3 Single‐factor experiment

The effects of PEG molecular weight, PEG concentration, ultrasonic power, enzyme concentration, temperature, and extraction time were examined by the method of single‐factor experiment. In detail, the extraction process was designed with a molecule weight of PEG (range from 200–1,000), PEG concentration (10%–50%), ultrasonic power (120–280 W), enzyme concentration (0.5%–2.5%), temperature (30–70°C), and extraction time (10–50 min). All experimental results were from three separate experiments.

### 2.4 Optimization of extraction conditions by BBD

According to the results of the single‐factor experimental data, a Box–Behnken design of three levels and three independent variables (PEG concentration, *X_1_*; ultrasonic power, *X_2_*; and extraction time, *X_3_*) was applied to investigate the influence on the extraction yield (*Y*, %) in this optimization study. Furthermore, each variable was set at three levels (high, medium, and low), coding + 1, 0, and −1, respectively. The BBD design was composed of 15 experiments, which include 12 factorial points and 3 center points in a random permutation (Table 1). A statistical software, named Design‐Expert, was used for data analysis by mathematical modeling and regression analysis. All experimental data were analyzed by multiple regressions to fit the following empirical second‐order polynomial model:

(1) $$Y=\beta_{0}+\sum_{i=1}^{3}\beta_{i}X_{i}+\sum_{i=1}^{3}\beta_{\mathrm{ii}}X_{i}^{2}+\sum_{i=1}^{3}\sum_{j=i+1}^{3}\beta_{\mathrm{ij}}X_{i}X_{j}$$

where *Y* is the response variable; *β_0_* is the constant; and *β_i_*, *β_ii_*, and *β_ij_* are the linear coefficient, quadratic coefficient, and the cross‐product coefficient, respectively. *X*_i_ and *X*_j_ are the different independent variables.

**Table 1:** Box–Behnken design and response values for the extraction yield of LJLP

Run	Extraction time (*X_1_*, min)	PEG concentration (*X_2_*, %)	Ultrasonic power (*X_3_*, W)	Extraction yield (*Y*, %)
1	1	0	−1	14.30
2	0	0	0	14.75
3	0	0	0	14.72
4	1	0	1	12.10
5	0	1	−1	13.67
6	1	−1	0	14.02
7	−1	−1	0	13.24
8	0	1	1	11.75
9	−1	0	−1	13.56
10	0	0	0	14.69
11	−1	1	0	13.49
12	1	1	0	13.48
13	0	−1	−1	13.61
14	−1	0	1	12.13
15	0	−1	1	12.24

### 2.5 Analysis of monosaccharide composition

The monosaccharide compositions of crud LJLP were measured by HPLC with procedure of previous report (Liu et al., 2017), which had a few modifications. 10 mg of LJLP was treated with 3 mol/L trifluoroacetic acid (TFA) at 100°C for 6 hr. After removal of TFA, other impurities were stripped from the residue by washing with methanol for 3 times and then redissolved in 2 ml water. After adding 0.2 ml 0.5 mol/L 1‐phenyl‐3‐methyl‐5‐pyrazolone (PMP) methanol solution, 0.2 ml 0.3 mol/L NaOH solution was mixed with the hydrolysate and incubated for 45 min at 65°C. The next step is the acid neutralization treatment of the mixture, followed by adding 1 ml trichloromethane and extracting for 3 times. The aqueous phase was collected to be determined by HPLC.

The determination process was carried out with an Agilent 1,260 HPLC system. The chromatographic conditions were as follows: Phenomenex Gemini C_18_ column (250 mm × 4.6 mm, 5 μm) with the temperature of 35°C; flow rate 0.8 ml/min; and detector wavelength 245 nm. As for the eluting solvents, it was a mixture of acetonitrile (82:18, v/v) and 0.05 M phosphate buffer (pH 6.8). The injection volume was 10 μl.

### 2.6 FT‐IR spectrometric analysis

The IR spectrum of crude LJLP was recorded with a Fourier Transform Infrared Spectroscopy (FT‐IR) (Bruker Equinox55, Germany) with the frequency range of 4000–400 cm^−1^. The sample was analyzed as KBr pellets.

### 2.7 Antioxidant activity analysis in vitro of LJLP

#### 2.7.1 Superoxide radical assay

The superoxide anion radical scavenging activity of LJLP was measured according to the previous report (Yang, Yang, Guo, Jiao, & Zhao, 2013) with a few modifications. 1.0 ml nitroblue tetrazolium (NBT), 1.0 ml nicotinamide adenine dinucleotide (NADH), and 0.4 ml phenazine methosulfate (PMS) were added to the sample solutions, respectively, and incubated for 5 min at room temperature, followed by determination at 560 nm. Ascorbic acid was used as standard. The formula of superoxide radical scavenging activity was as follows: Scavenging rate (%) = [1‐(Abs. of sample‐Abs. of blank)/Abs. of control] ×100.

#### 2.7.2 Total reducing activity

The total reducing power of LJLP was assessed according to a reported procedure (Sun, Yang, Lu, Wang, & Zhao, 2013). 2.5 ml 0.2 M phosphate buffer saline (pH 6.6) and 2.5 ml 1% (w/v) K_3_Fe(CN)_6_ solution were added to 1 ml sample solution and mixed completely. After incubation for 20 min at 50°C, the mixture was mixed with 2.5 ml of 10% (w/v) trichloroacetic acid (TCA) solution. 2 ml of the supernatant was collected by centrifugation, followed by adding 2 ml distilled water and 0.5 ml 0.1% (w/v) FeCl_3_. The sample was determined at 700 nm, using ascorbic acid as a standard.

### 2.8 Antioxidant activity analysis in vivo of LJLP

Male nude Kunming mice aged 30 days were used in this study. The animal experimental protocols were performed strictly in compliance with the Ethics Committee Guide of China Huanggang Normal University. The mice were randomly assigned into seven groups, with five mice per group. In the LJLP group, mice were administered with LJLP at four different doses (100, 200, 400, and 800 mg/kg, BW per day), and receiving daily 100 mg/kg d‐galactose hypodermically; in negative control group (NCG), mice were treated with daily 10 ml/kg physiological saline intragastrically and hypodermically; in model control group (MCG), mice received daily 100 mg/kg d‐galactose by hypodermic injection and daily 10 ml/kg physiological saline by gastric gavage; in positive control group (PCG), mice were administered with daily 100 mg/ kg ascorbic acid and hypodermically injected daily d‐galactose (100 mg/kg BW per day). The animal test was carried out once a day for 6 weeks.

After 24 hr of fasting, the mice were weighed and put to death by decapitation. Blood samples from orbital sinus were immediately treated for isolation of serum. The livers were then excised and weighed, followed by homogenized in a medium containing 0.2 M phosphate buffer (4°C, pH 7.4). After centrifugation for 10 min at 4°C, supernatant was obtained for biochemical analysis. Assay kits (Nanjing Jiancheng) were used to observe the content of superoxide dismutase (SOD), catalase (CAT), glutathione peroxidase (GSH‐Px), total antioxidant capacity (TAOC), and malondialdehyde (MDA) level in blood serum and liver.

## 3 RESULTS AND DISCUSSION

### 3.1 Single‐factor experimental analysis

#### 3.1.1 Effects of PEG molecule weigh on LJLP yield

**Figure 1:**
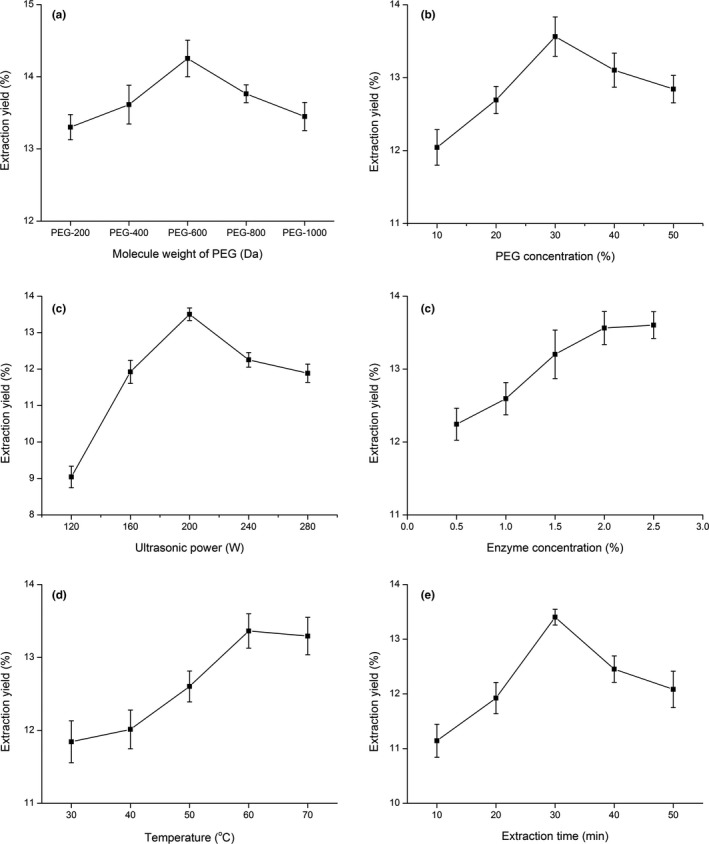
Effect of different extraction factors ((a) molecule weight of PEG; (b) PEG concentration, %; (c) ultrasonic power, W; (d) enzyme concentration, %; (e) extraction temperature, ^o^C; (f) extraction time, min) on the extraction yield of LJLP. The effects of molecule weight of PEG, PEG concentration, ultrasonic power, enzyme concentration, extraction temperature, and extraction time were first studied by a single‐factor design as follows: One factor was changed while the other factors were kept constant in each experiment. The effect of each factor was assessed by determining the extraction yield of LJLP

Obtaining the polysaccharides usually includes two steps: (1) Polysaccharides were extracted from raw materials with solvents; (2) the precipitation of polysaccharides from the extraction solution. PEG forms the hydrogen bond with polysaccharides, which could help to achieve higher solubility of polysaccharides. So PEG shows a positive effect on diffusion coefficients of extraction solvent (Zhou, Liu, Ma, & Zhang, 2014). In our study, PEG‐200, PEG‐400, PEG‐600, PEG‐800, and PEG‐1000 aqueous solutions (30%, v/v) were tested as extracting solvents of LJLP with UAEE, respectively. As shown in Figure 1a, a PEG‐600 aqueous solution supported the optimal yield of LJLP (14.25 ± 0.16%, *n* = 3). It may be explained by the fact that both the viscosity and polarity of PEG are closely related to its molecule weight (Zhou et al., 2011), and PEG‐600 can help to gain the maximal extraction yield of LJLP by providing a better solution condition, compared with other PEG aqueous solutions. Therefore, PEG‐600 was selected for the further studies.

#### 3.1.2 Effects of PEG concentration weigh on LJLP yield

To find suitable polarity of extraction solvent for the extraction of LJLP, different PEG concentrations were investigated in this study. As shown from Figure 1b, the extraction yields started to increase with increasing PEG concentration, and similar change rules of extracting polysaccharides from Ginkgo biloba leaves had been reported (Zhang, Zhang, et al., 2015) and reached a maximum at the 30%, then followed by an obvious decrease with further increases in PEG concentration. It was because that a relatively lower PEG concentration might support a more suitable viscosity of PEG aqueous solution, which may increase the efficiency of extraction and energy transferring in solution. Thus, the PEG‐600 concentration of 30% was the most suitable choice.

#### 3.1.3 Effects of Ultrasonic power on LJLP yield

Appropriate ultrasonic power is a benefit to improve the efficiency of disrupting cell wall, which has a crucial influence on the extraction rate of polysaccharide. The ultrasonic power in the UAEE was studied in the range from 120 to 280 W, while keeping the other extraction conditions at fixed levels. As can be seen in the Figure 1c, there was a significant increase in the extraction rate of LJLP with increasing ultrasound power from 120 to 200 W, which peaked at 200 W and then decreased. It may be the reason that proper ultrasound power could accelerate solvent penetration into the plant cells and benefit dissolution of the components to be extracted. But over high level of ultrasonic power may destroy the polysaccharide (Li et al., 2013; Zhang, Guo, Wang, & He, 2015). Therefore, the optimal ultrasonic power should be 200 W.

#### 3.1.4 Effects of enzyme concentration on LJLP yield

To enhance the efficiency of cell disrupting, which benefits polysaccharide releasing from the cell, complex enzymes (cellulose, pectinase, and papain) were used in this study. As shown in Figure 1d, the 2.0% (w/v) of enzyme concentration resulted in the maximal extraction yield of LJLP, which remained stable with a further increasing of concentration. A possible explanation is that the overdosed enzyme cannot bind to the substrates, so the hydrolysis rate slows down and has no positive effect of LJLP extraction yield. Therefore, the complex enzyme concentration of 2.0% (w/v) was chosen for the extraction of LJLP.

#### 3.1.5 Effects of extraction temperature on LJLP yield

The effect of different extraction temperatures (30, 40, 50, 60, and 70°C) on LJLP yield was investigated, while the other factors keeping constant at the center. Figure 1e revealed that increasing extraction temperature resulted in a significant increase in extraction yields up to 60°C, while further rising of temperature did not benefit extraction. It may be explained by the fact that the higher the extraction temperature, the smaller the viscosity of the extraction solvent, which could benefit penetration of extraction solvent into the broken cells and promote desorption of polysaccharides from the cell matrix, resulting in higher diffusion speed and solubility of polysaccharides in the extraction solvent (Chen, Li, Liu, Yang, & Li, 2012). Nevertheless, too high a temperature would cause the inactivation of enzyme (Yin, You, & Jiang, 2011) and degradation of polysaccharides (Chen, Tang, Chen, Wang, & Li, 2010), which disadvantage the extraction yield of polysaccharides. Thus, the extraction temperature of 60 ^◦^C was suitable for the study.

#### 3.1.6 Effects of extraction time on LJLP yield

To determine the shortest time for sufficient recovery for LJLP in a single extraction, the extraction time was assessed in this test. Figure 1f showed the influence of different extraction time on the extraction rate of LJLP. There was a significant increase of extraction yields with the time extended from 10 to 30 min. A longer time would lead to an obvious decrease of extraction yields, owing to degradation of polysaccharides caused by over excessive extraction time. In this single‐factor experiment, we chose 30 min as the best extraction time.

### 3.2 Optimization of the LJLP extraction

Based on the results of single‐factor experiment, three experimental factors (PEG concentration, ultrasonic power, and extraction time) were selected as the independent variables of BBD, while other factors (extraction temperature, enzyme concentration, and PEG molecule weight) were fixed at 60 ^◦^C, 2.0% (w/v), and PEG‐600, respectively. From BBD and experimental results of extraction yield of LJLP (Table 1), a second‐order polynomial regression model was as follows:

$$\begin{array}{c} Y=-28.89875+0.46125X_{1}+0.52925X_{2}+0.30781X_{3}-1.\text{975 e}-3X_{1}X_{2}\\ -4.\text{8125e}-4X_{1}X_{3}-3.\text{4375e}-4X_{2}X_{3}-4.\text{7875e}-3X_{1}^{2}-6.8375X_{2}^{2}-7.\text{61719e}-4X_{3}^{2} \end{array}$$

From the analysis of variance (ANOVA) results (Table 2), a *p*‐value < .0001 and an *F*‐value of 403.84 reveal that the model term has a high significance. The precision of regression model mainly depends on the coefficient of determination *R* ^2^, adjusted coefficient of determination *R* ^2^_Adj_ and coefficient of variance (C.V.). The *R* ^2^ value of 0.9986 shows there is strong relevance of the dependent variables in the model. The *R* ^2^_Adj_ value of 0.9962 also revealed that the adjusted model has a high significance. The coefficient of variation (C.V.) demonstrates the dispersion degree between predicted and experimental data, and C.V. of 0.46% for the model indicated a good dependability for experimental data and a high accuracy of the model. Considering “lack of fit *F*‐value” of 6.43 and its insignificance (*p* > .05), it means that the predicted model was in good agreement with experimental model.

**Table 2:** Analysis of variance (ANOVA) for the quadratic polynomial model

Source	Sum of square	*df*	Mean square	*F*‐value	*P*‐value
Model	13.92	9	1.55	403.84	<.0001*
*X*_1_	0.27	1	0.27	71.49	.0004*
*X*_2_	0.065	1	0.065	16/92	.0092*
*X*_3_	5.99	1	5.99	1562.87	<.0001*
*X*_1_ *X*_2_	0.16	1	0.16	40.74	.0014*
*X*_1_ *X*_3_	0.15	1	0.15	38.70	.0016*
*X*_2_ *X*_3_	0.076	1	0.076	19.75	.0067*
*X*_1_^2^	0.85	1	0.85	220.96	<.0001*
*X*_2_^2^	1.73	1	1.73	450.71	<.0001*
*X*_3_^2^	5.48	1	5.48	1,431.95	<.0001*
Lack of fit	0.017	3	5.783e−3	6.43	.1376
Pure error	1.8e−3	2	9.0e−4		
*R* ^2^ = 0.9986; Adjusted *R* ^2^ = 0.9962; CV = 0.46%.					

Abbreviation: *df*, degree of freedom.

* Means significant (*p* < .05).

It is well known that the model coefficient has a statistical significance considering that the value of “Prob > *F*” is less than 0.05 (Liu et al., 2013). Based on the level of *p*‐value, the linear variables *X*_3_, and the quadratic variables *X*_1_^2^, *X*_2_^2^, and *X*_3_^2^ were statistically very significant at *p* < .0001, whereas the linear variables *X*_1_ and *X*_2_, and two‐variable interaction *X*_1_ *X*_2_, *X*_1_ *X*_3_, and *X*_2_ *X*_3_ had significant influence (*p* < .05) on the extraction yield of LJLP. Therefore, three independent variables (PEG concentration, ultrasonic power, and extraction time) were all significantly associated with the extraction yield of LJLP.

**Figure 2:**
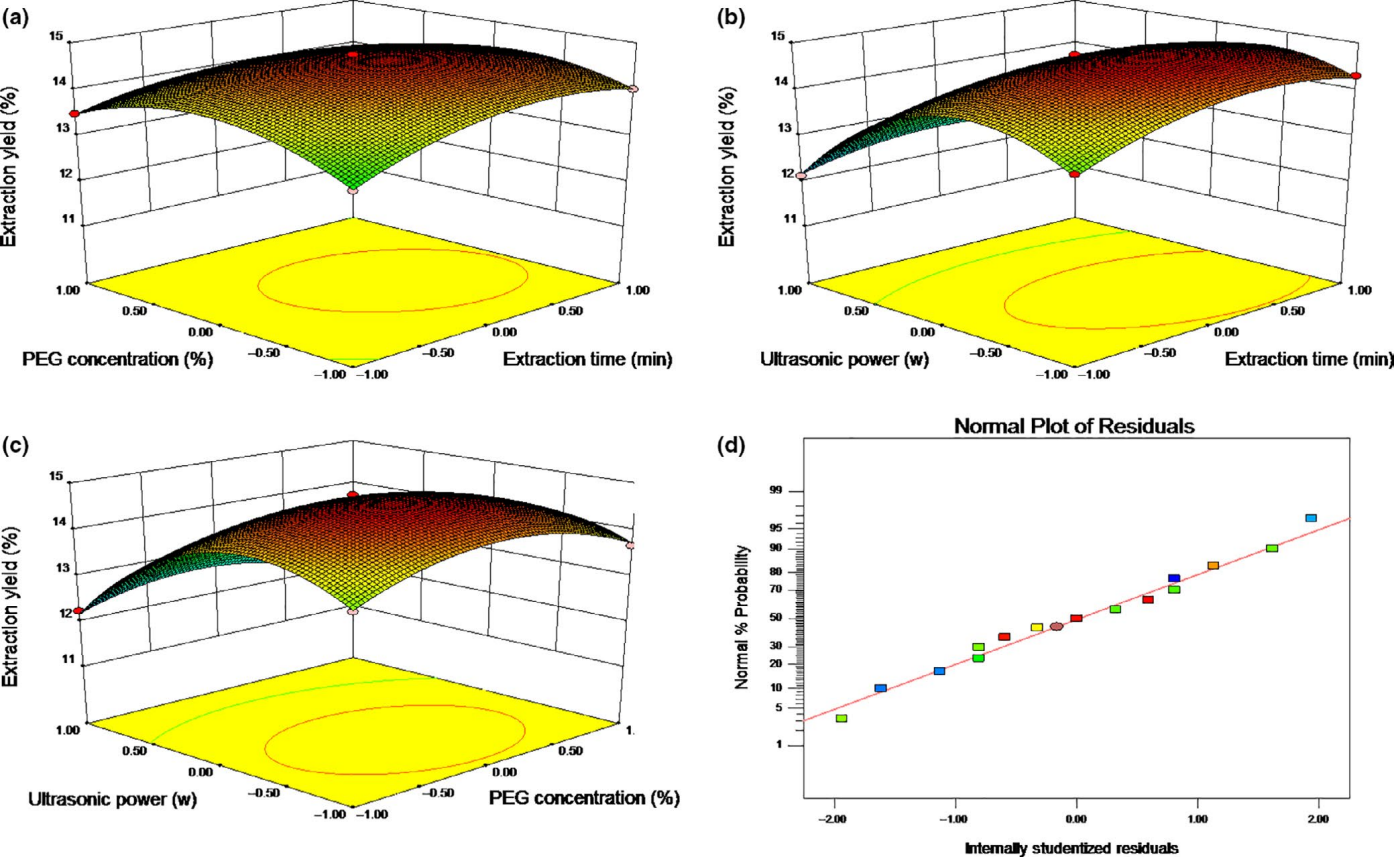
Response surface plots for the effect of independent variables on the extraction yield of LJLP

To uncover how experimental factors and their interactions affect the extraction rate, Design‐Expert was used to plot response surfaces (Zhang, Guo, et al., 2015). The results of extraction yield of LJLP influenced by extraction time (*X*_1_), PEG concentration (*X*_2_), and ultrasonic power (*X*_3_) are shown in Figure 2a‐d. It can be seen from Figure 2 that the response surface revealed that the extraction yield depended largely upon the PEG concentration, extraction time, and ultrasonic power. This was in perfect accordance with the results of the ANOVA.

According to the regression equation, the optimal extraction conditions were calculated by Design‐Expert software, which were an extraction time of 33.70 min, a PEG concentration of 30.15% and an ultrasonic power of 191.11 W. To verify the validity of the mathematical model, tests were performed in triplicate under the adjusted extraction conditions, which are extraction time (33 min), PEG concentration (30%), and ultrasonic power (191 W). An extraction yield of 14.76% was achieved under the adjusted experimental conditions, which were close to the predicted value (14.87%) of the model.

### 3.3 Chemical characterization of LJLP

#### 3.3.1 Monosaccharide composition by HPLC

**Figure 3:**
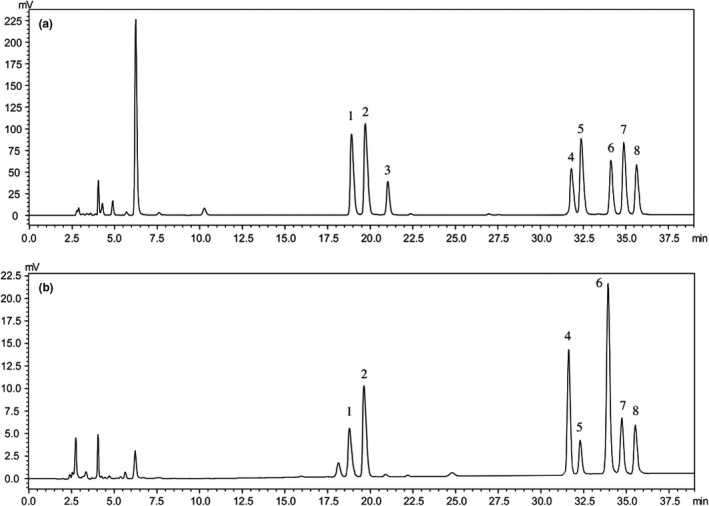
The HPLC chromatograms of 8 standard monosaccharides (a) and component monosaccharides released from LJLP (b). Peaks 1. mannose, 2. ribose, 3. rhamnose, 4. glucose, 5. xylose, 6. galactose, 7. arabinose, and 8. fucose

The monosaccharide compositions of crude LJLP were determined by the aid of HPLC (Figure 3). Crude LJLP contained mannose (8.2%), ribose (15.2%), glucose (20.9%), xylose (5.7%), galactose (32.3%), arabinose (9.5%), and fucose (8.2%), respectively. The composition analysis of LJLP clearly showed that the main monosaccharide was galactose with a molar percentage ratio of 32.3% among total compositional carbohydrates, followed by glucose (20.9%) and ribose (15.2%), totaled to 68.4%. Therefore, all results indicated that LJLP was a heteropolysaccharide.

#### 3.3.2 Infrared spectra analysis

**Figure 4:**
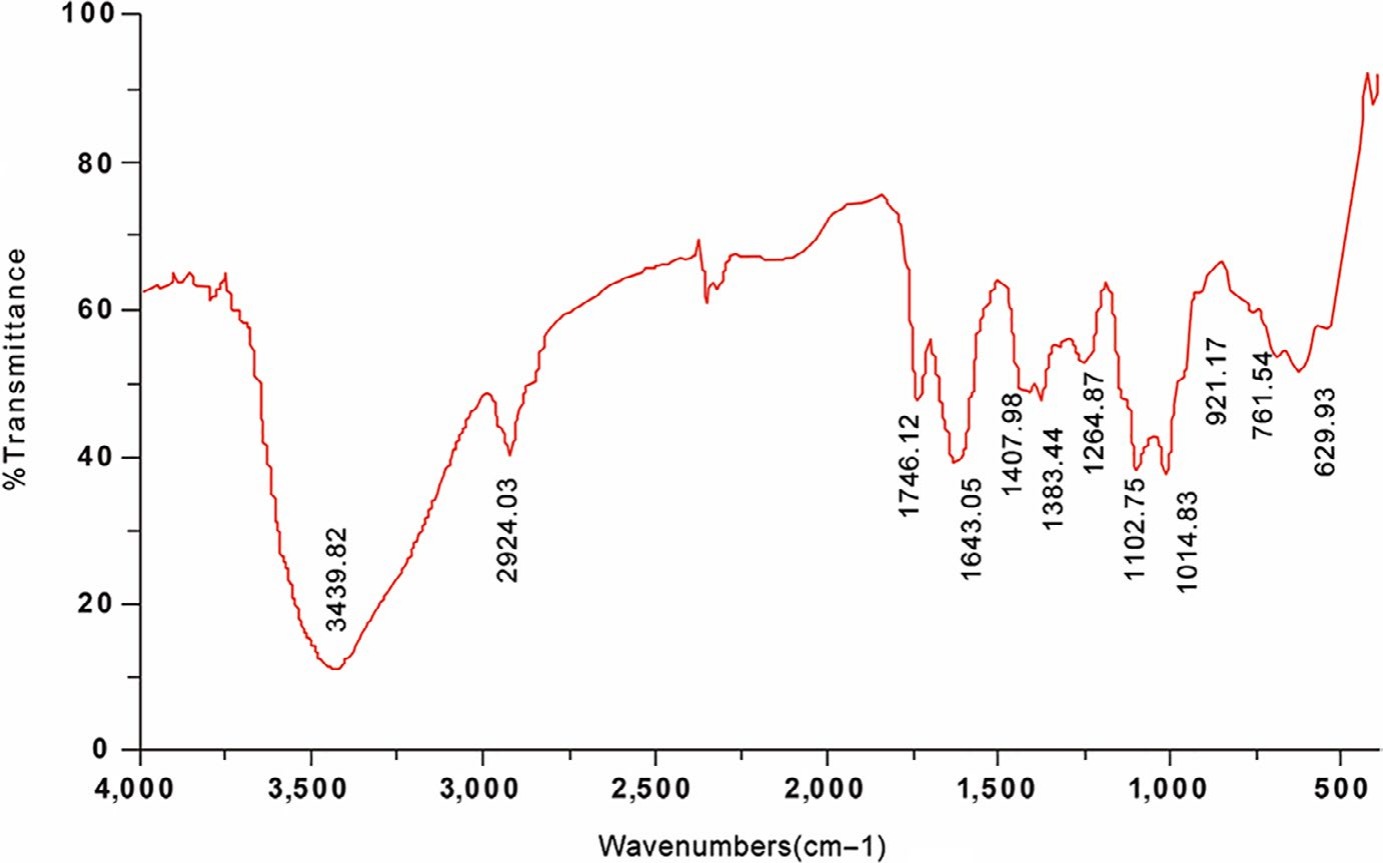
FT‐IR spectroscopy of LJLP between 4,000 and 400 cm^−1^

As shown in Figure 4, the fundamental groups in the structure of LJLP were determined by the aid of FT‐IR analysis. The strong absorption peak around 3,400 cm^−1^ was ascribed to the ‐OH stretching vibration, which may reveal intermolecular and intramolecular hydrogen bonds existing in the polysaccharide. The weak peak around 2,924 cm^−1^ was from C‐H stretching vibration. The peaks at around 1643 cm^−1^ and 1,407 cm^−1^ suggested the presence of ester carbonyl groups (C = O) and carboxylic groups (COO‐), supporting the fact that LJLP polysaccharide contained uronic acids (Al‐Sheraji et al., 2012). The absorption peaks at 1000–1,200 cm^−1^ suggested two types of C‐O stretching vibration, indicating that there were pyranose rings in the structure of LJLP polysaccharides (Du et al., 2016).

### 3.4 Antioxidant activities in vitro of LJLP

#### 3.4.1 Superoxide radicals scavenging activity

**Figure 5:**
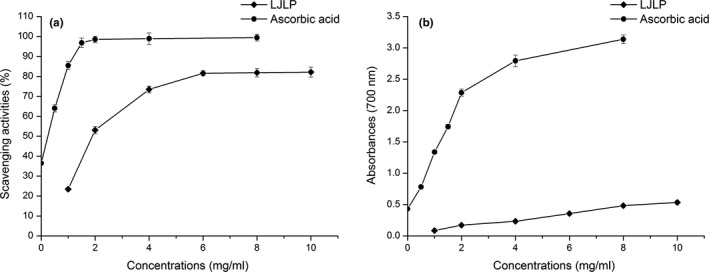
Antioxidant activities (a) scavenging effects on superoxide radical, (b) scavenging effects on total reducing power of LJLP

Superoxide radical has harmful effects on DNA and membrane lipid of the cell which may lead to cell damage (Macdonald, Galley, & Webster, 2003). The comparisons were made between LJLP and ascorbic acid based on their superoxide radical scavenging activity. As shown in Figure 5a, LJLP demonstrated that scavenging activity on superoxide radicals was in direct proportion to its concentration. LJLP at a concentration of 6 mg/ml supported the maximum scavenging ability (81.56%), while ascorbic acid could maximally take the scavenging activity (98.52%) at 2 mg/ml. In relatively low concentration, ascorbic acid exhibited much higher scavenging activity than LJLP. However, as concentration went up to 1.0 mg/ml, LJLP exhibited much stronger scavenging ability. The above results revealed that LJLP had a significant effect on scavenging superoxide radicals.

#### 3.4.2 Reducing power assay

In this experiment, the Fe^3+^/ferricyanide complex was reduced into the ferrous form in the presence of reducers (i.e., antioxidants). Thus, the Fe^2+^ concentration was determined by measuring the formation of Perl's Prussian blue at 700 nm (Jin et al., 2012). Figure 5b describes the relationship of the reducing power of LJLP versus their concentration. The higher the absorbance value, the higher the total reducing power of LJLP. It can be seen from Figure 5b that the total reducing power of LJLP gradually increased with the increasing concentration and had a good correlation with polysaccharide concentration. The reducing power of LJLP was much lower than ascorbic acid. It may be the reason that the ketones of LJLP react with the specific peroxide precursors, thus preventing the formation of peroxide (Elmastaş et al., 2006).

### 3.5 Antioxidant activities in vivo of LJLP

In this research, the antioxidant activity of LJLP in vivo was evaluated in a mice aging model, which was established by d‐galactose. As shown in Tables 3 and 4, the activities of CAT, SOD, GSH‐PX, and TAOC of the aging model group were significantly lower than those of the normal control group (NCG) in serum and liver, while the contents of MDA in serum and liver were significantly higher than those in the NCG. The above results suggested that establishment of the aging mice model was successful in this study.

**Table 3:** Effects of LJLP on the activities of CAT (U/ml), SOD (U/ml), GSH‐Px (U/ml), TAOC (U/ml), and levels of MDA (nmol/ml) in serums of aging mice

Groups	CAT	SOD	GSH‐Px	TAOC	MDA
NCG (saline)	36.58 ± 3.97	142.62 ± 7.46	3,611.29 ± 213.48	26.54 ± 1.79	66.25 ± 7.12
MCG (D‐Gal)	21.06 ± 4.25^a^	76.87 ± 3.27^a^	2,169.85 ± 174.28^a^	17.21 ± 2.41^a^	86.83 ± 6.67^a^
PCG (ascorbic acid)	33.26 ± 6.02^b^	134.45 ± 7.86^b^	3,830.23 ± 253.13^b^	24.25 ± 1.26^b^	69.23 ± 6.23^b^
LJLP (100 mg/kg)	23.14 ± 3.54	90.88 ± 4.48	2,615.43 ± 197.51^c^	20.14 ± 1.89^c^	81.60 ± 5.76^c^
LJLP (200 mg/kg)	28.64 ± 6.38^b^	113.27 ± 6.13^b^	3,111.42 ± 238.90^b^	22.03 ± 2.25^b^	77.35 ± 6.42^b^
LJLP (400 mg/kg)	36.16 ± 8.65^b^	139.78 ± 5.59^b^	3,678.35 ± 291.62^b^	24.84 ± 2.58^b^	70.60 ± 4.71^b^
LJLP (800 mg/kg)	42.31 ± 7.64^b^	162.15 ± 5.34^b^	3,958.49 ± 329.85^b^	28.18 ± 2.25^b^	63.35 ± 7.04^b^

Note

Values were expressed as mean ± *SD* (*n* = 5) and evaluated by one‐way ANOVA.

a *P* < .01 versus NCG.

b *P* < .01 versus MCG.

c *P* < .05 versus MCG.

**Table 4:** Effects of LJLP on the activities of CAT (U/ml), SOD (U/ml), GSH‐Px (U/ml), TAOC (U/ml), and levels of MDA (nmol/ml) in livers of aging mice

Groups	CAT	SOD	GSH‐Px	TAOC	MDA
NCG (saline)	27.09 ± 3.79	240.82 ± 8.64	515.15 ± 44.28	2.97 ± 0.19	18.70 ± 2.44
MCG (D‐Gal)	12.84 ± 2.27^a^	137.47 ± 11.65^a^	294.58 ± 31.88^a^	1.83 ± 0.08^a^	23.74 ± 1.76^a^
PCG (ascorbic acid)	26.13 ± 4.32^b^	224.38 ± 17.72^b^	498.93 ± 35.76^b^	2.92 ± 0.18^b^	19.23 ± 2.32^b^
LJLP (100 mg/kg)	22.08 ± 4.84^b^	148.36 ± 3.88	335.63 ± 16.41	2.43 ± 0.22^b^	21.62 ± 1.06^c^
LJLP (200 mg/kg)	23.72 ± 6.43^b^	187.72 ± 9.32^c^	384.42 ± 43.09^b^	2.62 ± 0.15^b^	20.15 ± 1.42^b^
LJLP (400 mg/kg)	26.46 ± 5.37^b^	231.64 ± 10.26^b^	437.53 ± 21.22^b^	2.93 ± 0.15^b^	18.06 ± 0.79^b^
LJLP (800 mg/kg)	30.11 ± 6.78^b^	256.24 ± 14.15^b^	469.39 ± 46.98^b^	3.18 ± 0.25^b^	17.13 ± 1.04^b^

Note

Values were expressed as mean ± *SD* (*n* = 5) and evaluated by one‐way ANOVA.

a *P* < .01 versus NCG.

b *P* < .01 versus MCG.

c *P* < .05 versus MCG.

In recent years, more and more evidence showed that the development of aging was largely due to the cumulative damage, which was caused by intracellular reactive oxygen species (ROS) (Jing et al., 2016). As the most important antioxidant enzymes, CAT, SOD, and GSH‐Px can inhibit the formation of free radicals and act as biomarkers to indicate the generation of ROS. It was revealed in Tables 3 and 4 that effects of LJLP on the activities of CAT, SOD, and GSH‐Px in serum and liver of aging mice. For both LJLP groups and ascorbic acid treatment group (PCG), CAT, SOD, and GSH‐Px activity was increased in serum samples, and the same with the liver. While treated with ascorbic acid and LJLP at the doses of 200, 400, or 800 mg/kg, the activities of these antioxidant enzyme were significantly higher than MCG, which means that both LJLP and ascorbic acid have a positive effect on CAT, SOD, and GSH‐Px activity of serum and liver samples.

It is known that the performance of the nonenzymatic ROS defense system could be evaluated by TAOC. According to Tables 2 and 3, LJLP‐treated groups and PCG supported a remarkable rise of TAOC activity in serum and liver of aging mice, showing that the nonenzymatic ROS defense system of aging mice had been improved. Since there is a direct relationship between generation of MDA and lipid peroxidation, the level of MDA acts as an oxidative stress marker. According to Tables 3 and 4, all LJLP‐treated groups and PCG exhibited significantly decreased MDA level both in serum and liver, indicating that LJLP could lead to a deceleration of lipid peroxidation in aging mice. The above results showed that LJLP had obvious roles in antioxidation and senescence resistance.

## 4 CONCLUSION

An environmentally friendly method was developed for the extraction of polysaccharides from *Lonicera Japonica* leaves. As a green solvent, PEG not only leads to less residual toxin left in the polysaccharides product, but also enhances the efficiency of flavonoid extraction and diminishes production costs. It had maximal extraction yield of 14.76% with optimized extraction conditions of LJLP: extraction time 33 min, PEG concentration 30%, and ultrasonic power 191 W. Moreover, The FT‐IR spectra and antioxidant activities of LJLP were evaluated. These results revealed that LJLP might have a great potential for application as an effective component of medicine or functional food ingredient.

## CONFLICTS OF INTEREST

The authors declare no conflict of interest.

## ETHICAL APPROVAL

All the animal experimental protocols were performed strictly in compliance with the Ethics Committee Guide of China Huanggang Normal University.
